# NMR Studies of the C-Terminus of alpha4 Reveal Possible Mechanism of Its Interaction with MID1 and Protein Phosphatase 2A

**DOI:** 10.1371/journal.pone.0028877

**Published:** 2011-12-14

**Authors:** Haijuan Du, Michael A. Massiah

**Affiliations:** Department of Chemistry, George Washington University, Washington, D.C., United States of America; University of South Florida College of Medicine, United States of America

## Abstract

Alpha4 is a regulatory subunit of the protein phosphatase family of enzymes and plays an essential role in regulating the catalytic subunit of PP2A (PP2Ac) within the rapamycin-sensitive signaling pathway. Alpha4 also interacts with MID1, a microtubule-associated ubiquitin E3 ligase that appears to regulate the function of PP2A. The C-terminal region of alpha4 plays a key role in the binding interaction of PP2Ac and MID1. Here we report on the solution structure of a 45-amino acid region derived from the C-terminus of alpha4 (alpha45) that binds tightly to MID1. In aqueous solution, alpha45 has properties of an intrinsically unstructured peptide although chemical shift index and dihedral angle estimation based on chemical shifts of backbone atoms indicate the presence of a transient α-helix. Alpha45 adopts a helix-turn-helix HEAT-like structure in 1% SDS micelles, which may mimic a negatively charged surface for which alpha45 could bind. Alpha45 binds tightly to the Bbox1 domain of MID1 in aqueous solution and adopts a structure consistent with the helix-turn-helix structure observed in 1% SDS. The structure of alpha45 reveals two distinct surfaces, one that can interact with a negatively charged surface, which is present on PP2A, and one that interacts with the Bbox1 domain of MID1.

## Introduction

Alpha4 was initially identified as an immunoglobin binding protein (Ig-αBP) involved in signal transduction in mammalian B and T lymphocytes [1], [2], [3], [4], [5]. Alpha4 and its yeast counterpart Tap42 were later shown to be key regulators of protein phosphatase 2A (PP2A) within the target of rapamycin (TOR) signaling pathway [3], [4], [6], [7], [8], [9], [10], [11]. Alpha4 binds to the catalytic subunit of PP2A (PP2Ac) and displaces the scaffolding and regulatory subunits that typically make up the PP2A heterotrimeric complex [3], [12], [13], [14], [15]. Under growth-promoting conditions TOR phosphorylates alpha4, which then binds and down-regulates the function of PP2Ac [4], [11]. The resulting downstream activations of translation initiation factor 4E and S6 kinase initiate cell cycle progression [4], [11]. In rapamycin-sensitive cells, rapamycin promotes dissociation in the alpha4–PP2Ac complex [4].

Alpha4 also interacts with MID1, a microtubule-associated ubiquitin E3 ligase of the TRIM protein family [16]. MID1 is postulated to facilitate the ubiquitination of PP2Ac [17], [18], [19], [20]. Mutations of MID1 result in X-linked Opitz Syndrome (XLOS) that is characterized by clefts lip/palate, wide-spaced eyes, and hyperspadias [17], [21], [22], [23], [24], [25], [26], [27]. Alpha4 co-expresses with MID1 along the same tissues affected by XLOS [18], [28]. Mutations of alpha4 result in overlapping MID1 birth defects [29], [30].

Alpha4 is postulated to be an important regulator of the function of MID1 and PP2Ac. The MID1–alpha4–PP2Ac complex results in either the dephosphorylation of MID1 and its dissociation from the microtubules or the ubiquitination of PP2Ac [17], [25]. Overexpression of alpha4 results in the redistribution of MID1 from the microtubule to the cytoplasm, suggesting that an increase in the alpha4-PP2Ac complex promotes dephosphorylation of MID1 [17]. Deletion of the alpha4 gene locus results in an increase in PP2Ac, indicating that the MID1-alpha4 complex is important for PP2Ac turnover.

The X-ray structures of the N-terminal fragment of alpha4 (residues 1–221) and the related Tap42 (residues 1–234) were recently determined [31], [32]. The structures were shown to be predominantly α-helical [31], [32]. Attempts to clone full-length alpha4 and Tap42 have resulted in truncated proteins with an N-terminal region corresponding to the amino acids noted above [33]. While residues 94–202 of alpha4 are important for PP2Ac binding, yeast two-hybrid studies indicated that deletion of the C-terminal domain (236–339) dramatically decrease the binding to PP2A [4]. In addition, the C-terminal domain of alpha4 is responsible for binding the Bbox1 domain of MID1 [25], . Thus, the C-terminus is important for the overall function of alpha4, and its structural characterization is important to gain insights into the mechanism of function of alpha4. Within the C-terminal domain of alpha4, a 45-amino acid region encompassing residues Glu236 to Leu280 was shown to bind tightly to MID1 [34].

Here, we report on the NMR structures of the 45-amino acid peptide (alpha45) in aqueous solution and in the presence of 1% sodium dodecyl sulfate (SDS). These results suggest that the C-terminus of alpha4 can undergo conformational changes based on protein-protein interaction or its environment.

## Materials and Methods

### Cloning and purification

Native human *alpha4* cDNA was used as the template to amplify the region encoding residues Glu236 to Leu280 (alpha45). The PCR fragment was cloned into the pMAL28 plasmid (New England Biolabs, Inc) as a fusion protein with an N-terminal His6-maltose-binding protein (MBP). The fusion protein is separated from alpha45 by a tobacco etch virus protease (TEV) cleavage site. The orientation and integrity of the vector were verified by DNA sequencing prior to transformation into BL21(*DE3*) cells (Lucigen Corp).

To obtain ^15^N and ^15^N/^13^C-labeled His_6_-MBP-alpha45, cells were grown in M9 minimal medium supplemented with ^15^N-ammonium chloride (^15^NH_4_Cl)/^12^C-glucose or ^15^NH_4_Cl/^13^C-glucose, respectively. The cells were initially grown at 37°C to an optical cell density of 0.6 and induced with 1 mM isopropyl-β-d-thiogalactopyranoside (IPTG) for 12 hrs at 22°C. Cells were harvested and stored at −80°C. For lysis, the cells were suspended in 50 mM Tris-HCl buffer (pH 8), with 25% (w/v) sucrose, phenylmethylsulfonyl fluoride (PMSF), protease inhibitors, lysozyme (1 mg/ml), magnesium sulfate (0.5 mM), DNase (0.2 mg/ml), 1% Triton-X 100 and 10 mM dithiothreitol (DTT) at 4°C, and pulse sonicated. The lysate was centrifuged at 20,000×*g* and the clarified supernatant was applied to the Ni^2+^-NTA affinity resin (Qiagen Inc.). The fusion protein was eluted with 50 mM Tris–HCl (pH 8) containing 200 mM imidazole, cleaved with TEV, and the peptide was purified by gel filtration.

### NMR experiments

Two sets of NMR samples were prepared, one in which the peptide was dissolved in aqueous solution and the other in which 1% SDS was added. The samples contained ∼0.5 mM ^15^N- and ^15^N-^13^C-labeled alpha45 in 50 mM Tris-HCl (pH 7.4), 0.2% sodium azide in 90% H_2_O/10% D_2_O (with and without 1% SDS). Data were acquired with Varian Inova 600 MHz spectrometer equipped with a 5 mm triple resonance (^1^H, ^13^C, and ^15^N) probe with *z*-axis gradient. Double and triple resonance NMR experiments were performed at 10°C for the aqueous samples. At higher temperatures (>10°C) most of the NMR signals in the HN-HN and HN-side-chain proton regions of the nuclear Overhauser effect spectroscopy (NOESY) spectrum were missing, most likely due to exchange of the NH protons with water. The data for the sample in 1% SDS were acquired at 21 °C instead of 10°C because the signals were sharper. Three-dimensional (3D) ^1^H-^15^N-NOESY-heteronuclear single quantum coherence (HSQC), and ^1^H-^13^C-HSQC-NOESY were acquired to identify ^1^H-^1^H sequential and long range nuclear Overhauser effects (NOE) correlations using a 150 millisecond mixing time. The 3D-^1^H-^15^N HSQC-TOCSY (50 msec) and ^1^H-^13^C HCCH-TOCSY (15 msec) data were acquired to assign through-bond correlations. The 3D HNCACB and HNCA spectra were acquired to obtain backbone heteroatom through-bond correlations.

The NMR data were processed with nmrPipe [35] and analyzed with SPARKY3 [36]. NOEs were grouped as strong, medium, or weak based on their intensities in the 3-D ^15^N- and ^13^C-edited NOESY spectra and assigned distance ranges of 1.8–2.8 Å, 1.8–3.3 Å, and 1.8–5.0 Å, respectively. For alpha45 in 1% SDS, hydrogen-bond restraints between the carbonyl and the amide groups were defined with two distance restraints (O-H, 1.8–2.5 Å and O-N, 2.7–3.5 Å). Hydrogen bonds were established from NOE patterns and the proximities of donor and acceptor groups in initial structures calculated with only NOE-derived restraints. The chemical shifts of the ^1^Hα, HN, ^15^N, ^13^Cα and ^13^Cβ atoms were input into TALOS+ [37] for dihedral angle estimation. The structures of alpha45 in aqueous solution and in 1% SDS were calculated with CYANA 2.1 [38]. The number of restraints used in the structure calculations is listed in Table 1. From a total of 50 randomly calculated structures, 14 structures were selected based on low target functions (tf<1), and low root mean standard deviation (RMSD) values (<1 Å) for backbone and heavy atom superposition. A total of 20,000 steps for torsion angle simulated annealing were employed. The quality of the structures was evaluated with Procheck [39].

**Table 1:** NMR Structure Determination Statistics.

	Alpha45 in H_2_O	Alpha45 in 1% SDS
**A. Restraint Statistics**		
Intra-residue NOEs	82	163
Sequential NOEs (|i -j| = 1)	42	147
Medium range NOEs (2≤|i -j|≤4)	58	88
Long range NOEs (|i -j|>4)	0	22
Total NOE restraints	182	416
Hydrogen bonds restraints	0	36
Dihedral angle restraints	58	62
Total Restraints	182	515
**B. Ensemble Statistics Analysis**		
*RMSD values (Å)*		
Backbone heavy atoms (helix I^a^)	0.26±0.09^b^	0.17±0.08
All heavy atoms (helix I)	1.26±0.17	0.67±0.13
Backbone heavy atoms (helix II^c^)	0.37±0.11^d^	0.21±0.07
All heavy atoms (helix II)	1.28±0.21	0.95±0.12
Backbone heavy atoms of helices I and II	n/a	0.38±0.10
All heavy atoms	n/a	0.93± 0.12
*Statistics from Ramachandran plot* e		
Residues in most favored regions (%)	88	83
Residues in additional allowed regions (%)	11	17
Residues in generously allowed regions (%)	0.7	0
Residues in disallowed regions (%)	0.3	0

a *Helix I in SDS includes residues 243 to 254.*

b *Helix I in H_2_O includes 247–253.*

c *Helix II in SDS includes residues 264 to 277.*

d *Helix II in H_2_O includes residues 266 to 276.*

e *Value averaged for 14 structures.*

To understand how the peptide interacted with the SDS micelles, MnCl_2_ was titrated into 0.4 mM ^15^N-labeled alpha45 at increments of 0.2 mM to a final concentration of 0.8 mM. HSQC spectra were acquired at each increment. Amino acids that were solvent exposed or could be in contact with Mn^2+^ would have their NMR signals broadened and disappear due to the paramagnetic effect of the Mn^2+^ ions, while those that interact with or may be embedded in the micelles will have their signals protected. As control, the titration was performed with alpha45 in aqueous solution.

### Bbox1-alpha45 Binding Studies by NMR

To determine whether Bbox1 and alpha45 interacted in aqueous solution and to identify the residues involved in the interaction, ^1^H-^15^N HSQC spectra were acquired at 21°C. The data were acquired at 21 °C instead of 10 °C because at the lower temperature Bbox1 would aggregate causing the NMR signals of both Bbox1 and Bbox1 bound alpha45 to broaden. First, 0.3 mM unlabeled (^1^H, ^14^N, ^12^C) Bbox1 was added to 0.3 mM ^15^N-labeled alpha45. Second, unlabeled 0.15 mM alpha4 was added to 0.3 mM ^15^N-labeled Bbox1. The Bbox1 domain was purified as described previously [40].

## Results

### Alpha45 is labile in aqueous solution

**Figure 1:**
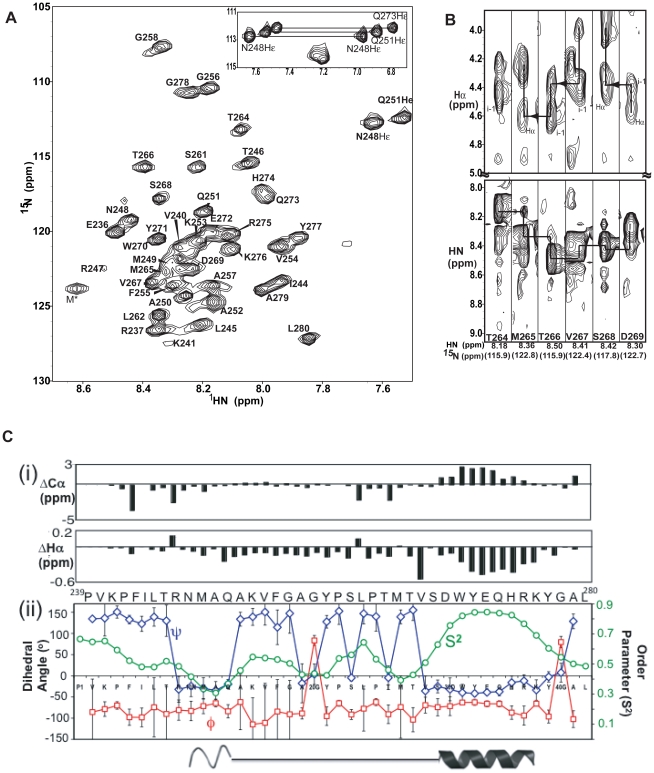
Secondary structure analysis of alpha45 in aqueous solution by NMR. **A.** HSQC spectrum of alpha45 in aqueous solution reveals that the NH signals fall within 1 ppm of each other, suggesting a labile structure. The NH assignments were identified using the 3D NMR data. M* represents one of the three amino acids that is the result from TEV cleavage; this residue is not part of alpha4 sequence. **B.** Strip plots taken from the 2D ^1^H-^1^H projections from the ^15^N planes of the 3D ^1^H-^15^N NOESY-HSQC spectrum showing NOE correlations between the NH_(i)_ to intra-residue and preceding Hα atoms (top panels) and sequential NH to NH atoms (bottom panels) for residues predicted to be helix II. An attempt to show NH-NH_(i,i±1)_ NOE correlations is indicated by lines, but the NOEs are weak, ambiguous and mostly missing. **C(i).** Analysis of the Cα and Hα atom chemical shift index (CSI) of alpha45 in aqueous solution. Upfield shifted Cα and simultaneous downfield shifted Hα values are indicative of α-helices. For the peptide in aqueous solution, helix I cannot be definitively characterized because while the Hα values are upfield shifted, the Cα values are closer to zero compared to those of helix II. **C(ii).** The phi (Φ, blue line) and psi (Ψ, red line) values and the order parameter (S^2^, green line) are plotted for each amino acid. These values were predicted by TALOS+ based on the chemical shift data. Residues adopting helical structure will have similar Φ and Ψ values. The higher the S^2^ values, the less mobile it is for that amino acid.

The two-dimensional ^1^H-^15^N HSQC spectrum of alpha45 in aqueous solution showed narrow dispersion (∼1 ppm) of the NH resonances, consistent with either a random coil or helical structure (Figure 1A). Peaks representing 40 of the 45 amino acids were observed and assigned to their corresponding amino acids using three-dimensional NMR data; there were five proline residues, which do not have N-H peaks. Sequential backbone ^1^H-^1^H and ^1^H-Cα_(i)_-Cα_(i-1)_ atom correlations were made using the 3D ^15^N-HSQC-NOESY and HNCA spectra, respectively. Even though this was a 45-amino acid peptide, which should give good through-band correctly, the ^1^H-CαCβ_(i)_-CαCβ_(i-1)_ correlations from the 3D HNCACB spectrum were incomplete. Only ^1^H-Cα_(i)_-Cα_(i-1)_ correlations were observed, similar to those of the HNCA spectrum. The HN-side-chain ^1^H-^1^H correlations observed with the^15^N-edited NOESY spectrum and ^1^H-Cα_(i)_-Cα_(i-1)_ connectivities with the HNCA spectrum were sufficient to identify the amino acids.

Alpha45 was predicted to contain two α-helices between residues Ile244 to Val254 and Val267 to Tyr277. Interestingly, there were no strong intensity sequential HN-HN_(i,i±1)_ NOEs, representing amino acids in helical conformations, observed for any regions of the peptide (Figure 1B, bottom). At best, the HN-HN_(i,i±1)_ NOEs were very weak and ambiguous, as depicted by possible cross-peaks connected by lines within the NH region in Figure 1B (bottom). Instead, strong intensity sequential ^1^HN-Hα_(i,i−1)_ NOEs representing amino acids in an extended or β-strand conformation were observed for the majority of the amino acids (Figure 1B, top). Aside from the intra-residue HN to side-chain proton NOE correlations, there were no medium or long-range inter-residue (|i-j|>3) NOEs, indicating that the peptide was highly extended, unstructured or highly labile. No side-chain to side-chain proton NOEs were observed with the 3D ^13^C-NOESY HSQC spectrum. Identification of ∼90% of the side-chain protons was made using the NOE cross-peak pattern from the ^13^C- and ^15^N-NOESY spectra and the ^15^N-edited TOCSY spectrum. The Cβ chemical shifts were identified from intra-residue ^1^H-^1^H cross peak patterns from the ^13^C-edited NOESY spectrum. In order to determine whether the lack of sequential or long range NOEs from the ^13^C- or ^15^N-NOESY spectra was due to incomplete NOE correlations, 2D ^1^H-^1^H NOESY spectra were acquired at various mixing times ranging from 50 to 800 ms. However, no NOEs indicating medium and long range side-chain ^1^H-^1^H interactions were observed.

### CSI and TALOS+ reveal presence of an α-helix in aqueous solution

Chemical shift indices (CSI) of backbone atoms can identify secondary structural elements [41], [42], [43]. The chemical shift values of the NH, N, Hα, Cα and Cβ atoms of non-proline amino acids of alpha45 were compared to their random coil values [44], [45]. While the CSI of Cβ and NH atoms can be consistent with protein secondary structures, the CSI of Cα and Hα atoms are more robust for identifying secondary structural elements [41], [43]. Thus, based on the CSI, the presence of one α-helical segment was indicated (Figure 1C(i)). For the residues predicted to be the first α-helix (residues 243–254), only the Hα chemical shifts showed larger deviation from random coil values, while those of the Cα and Cβ resonances were much smaller (<0.4 ppm). In contrast, the CSI values of both the Hα and Cα atoms for residues predicted to be helix II (residues 264–276) were consistent with the values observed for α-helices (Figure 1C(i)). The CSI values for the Cα atoms were in the range of 3 ppm greater than random coil values.

Chemical shift values of the NH, N, Cα, Cβ, Hα, and Hβ atoms were input into TALOS+ to estimate the phi and psi dihedral angles. TALOS+ uses a neural network that pre-classifies the secondary structure into alpha and beta conformations and calculates the random coil index (RCI) ‘order parameter’ to identify residues with substantial dynamics. TALOS+ predicted dihedral angles consistent with an α-helix for residues Thr264 to Lys276 (Figure 1C (ii)). The predicted phi (Φ) and psi (Ψ) values were plotted for each amino acid. Regions in which the Φ and Ψ values are similar indicate α-helical structures. The order parameters (RCI) representing the mobility of the N-H bond vector were plotted for each amino acid (Figure 1C(ii)). Interestingly, residues Arg247 to Ala252 had Φ and Ψ values consistent with an α-helix, but their RCI values were shown to be ∼0.45±0.2, indicating these residues were highly labile and may not exist in an α-helix in solution. In contrast, residues Val267 to Lys276 had Ψ and Φ values consistent with an α-helix and RCI values ranging from 0.7 to 0.9, suggesting that the C-terminal helix may exist in a relatively stable but transient state in aqueous solution.

### Alpha45 adopts two stable α-helices in 1% SDS

**Figure 2:**
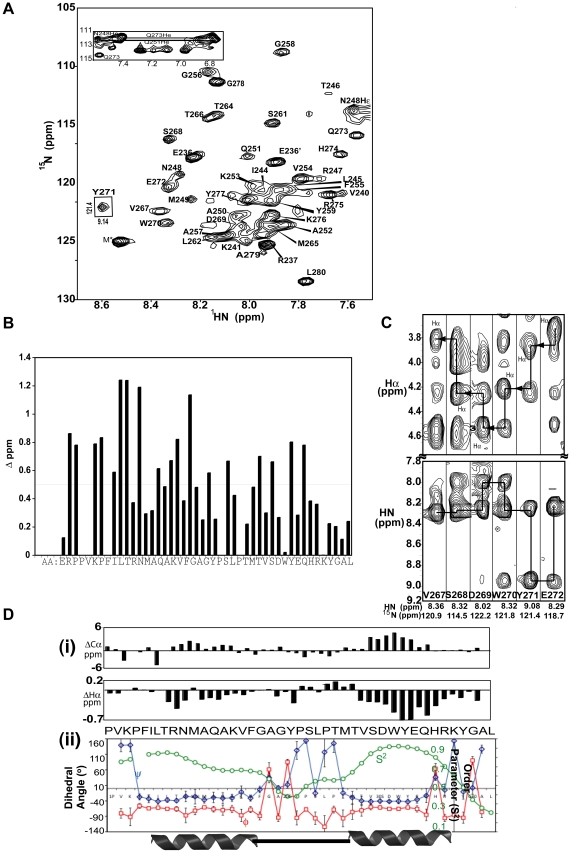
Secondary structure of Alpha45 in 1% SDS. **A.** HSQC spectrum of alpha45 in 1% SDS shows changes in chemical shifts for ∼60% of the amino acids, most notably Tyr271 (boxed). The peaks are labeled to their corresponding amino acids based on assignments using the 3D NMR data. **B.** Chemical shift differences of the NH and ^15^N atoms for alpha45 in 1% SDS compared to aqueous solution are shown to indicate amino acids that were most sensitive to the presence of SDS. The NH and ^15^N shifts are normalized to the equation [((ΔδNH)^2^ + (0.2*ΔδN)^2^)^1/2^]. Larger chemical shift changes were observed for residues within the first half of alpha45, based on Δδ>0.5 p.p.m. **C.** Strip plots taken from the 2D ^1^H-^1^H projections from the ^15^N planes of the 3D NOESY HSQC spectrum show strong intensity NH-NH_(i,i±1)_ NOEs for residues Val267 to Glu272 in 1% SDS. These residues are part of helix II. The corresponding NH_(i)_ to intra-residue and preceding Hα atoms are also shown (top panels). Compared to Figure 1B, the sequential NH-NH signals are dispersed, strong, and unambiguous. **D(i).** The CSI of the Cα and Hα atoms of alpha45 in 1% SDS reveal upfield shifted Cα and simultaneous downfield shifted Hα values, indicative of two α-helices. **D(ii).** The phi (Φ, blue line) and psi (Ψ, red line) values and the order parameter (S^2^, green line) are plotted for each amino acid. The Φ and Ψ values are consistent with the CSI values, while the S^2^ values, in the range of 0.7 to 0.9, indicate stability for the two helices.

With the observation that alpha45 was structurally labile in aqueous solution but may possess helical segments, the peptide was subjected to different conditions to determine whether its secondary and tertiary structures could be stabilized. SDS was found to be the best stabilizer. Previous reports have shown that SDS can induce or stabilize structures of short peptides [46],[47]. Upon adding 1% SDS to the NMR sample, large chemical shift changes were observed with the ^1^H-^15^N HSQC spectrum (Figures 2A, B). Normalizing the NH and ^15^N shifts to the equation [((ΔδNH)^2^ + (0.2*ΔδN)^2^)^1/2^] [48], greater chemical shift changes were observed for residues within the N-terminal half of the peptide (Val240 to Lys253, Figure 2B). The stability of the peptide in 1% SDS was confirmed by raising the temperature of the sample from 21 to 37°C and monitoring the structure by HSQC spectroscopy. After re-referencing the peak positions to account for temperature effect, the chemical shifts of all the amino acids remained unchanged, indicating the structure of the peptide was stable in 1% SDS. At the lower temperature (10°C), the resonances became broadened due to slower tumbling rate of the SDS-peptide complex and not conducive for NMR analysis.

Similar to alpha45 in aqueous solution, the NMR signals of 40 of the 45 amino acids were assigned. Strong intensity HN-HN_(i±1,2,3)_ and weaker HN-Hα_(i–>1−4)_ NOEs, indicative of helical structures, were observed for residues Phe243 to Phe255 and Thr264 to Tyr277 (Figure 2C). Compared to alpha45 in aqueous solution (Figure 1B), the HN-HN_(i,i±1)_ proton NOEs for residues Thr264 to Tyr277 were more dispersed and significantly stronger in intensity (Figure 2C). CSI and dihedral angle values estimated with TALOS+ were consistent with the NOE-derived secondary structural elements (Figure 2D(i, ii)). For residues of both helices, the RCI values were >0.7, indicating stable secondary structures (Figure 2D (ii)).

**Figure 3:**
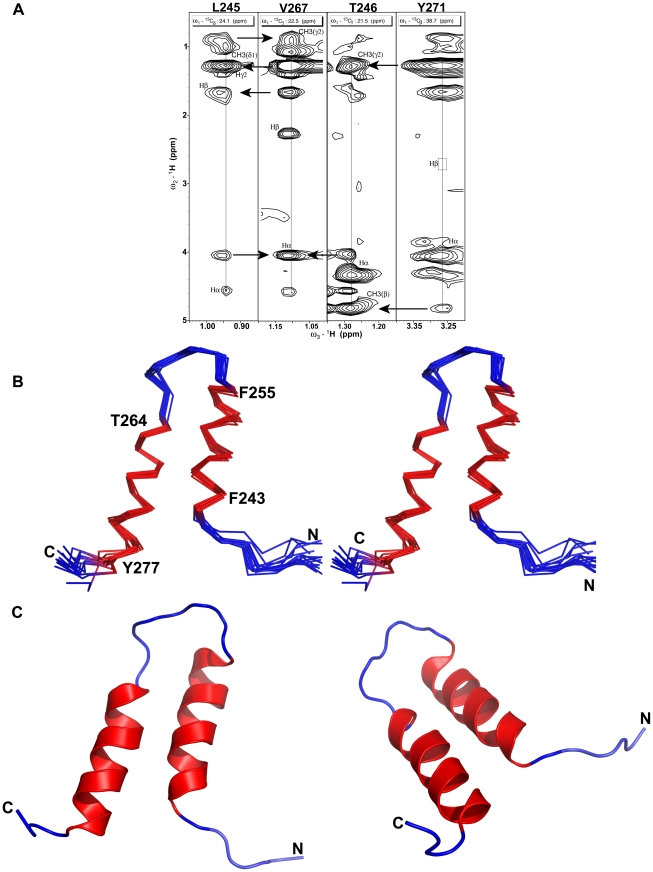
Structure of alpha45. **A.** Strip plots taken from the 2D ^1^H-^1^H projections of the ^13^C planes of the 3D-^1^H-^13^C-edited HSQC-NOESY show long range NOEs between residues Val267 with Leu245 and Thr246, and between Thr246 and Tyr271, which were used in the tertiary structural calculation of alpha45. All intra-residue NOEs are labeled on the figure and arrows indicate inter-residues NOEs. In this modified version of the ^13^C-edited NOESY spectrum, the diagonal auto-peaks were suppressed. **B.** Superposition of the backbone Cα, C, N atoms for residues 243 to 277 of fourteen structures of alpha45 calculated based on NMR restraints acquired in 1% SDS. The backbone atoms of the two α-helices are colored red (see Table 1). **C.** Ribbon representation of the structure of alpha45 in 1% SDS. For clarity, two orientations of the structure are shown using the same color scheme noted above.

In contrast of alpha45 in aqueous solution for which the NMR data showed a lack of side-chain to side-chain proton NOEs, the ^1^H-^13^C-HSQC-NOESY data revealed numerous long range (|i-j|>4) NOEs. The position of the two helices with respect to each other was defined by 22 non-redundant long-range NOEs between 11 residues (Figure 3A).

### Structure description of alpha45

A summary of the restraint input data and statistics of the calculated structures are listed in Table 1. The structures with the lowest target function values (<1.0), NOE violations <0.15 Å, dihedral angles <2°, and van der Waals violations <0.2 Å were selected. For alpha45 in aqueous solution, the ensemble of structures generated did not yield tertiary structures that were superimposable, even though restraints for strong intensity NH-Hα_(i−1)_ NOEs were used in the initial calculation. When dihedral angles for 29 amino acids were added to the calculation, two α-helices were defined for residues Arg247 to Val254 and Thr266 to Lys276 (structure not shown). Superposition of backbone atoms for N- and C-terminal helices resulted in root-mean square deviation (RMSD) values of 0.37±0.11 and 0.26±0.09 Å, respectively (Table 1). Since the peptide may be labile in aqueous solution, the structure may simply reflect a sampling of the actual conformation of the peptide as it undergoes folded-unfolded transition. The relative position of the two helices could not be defined due to lack of long range NOEs.

The calculated structures of alpha45 based on restraints acquired in 1% SDS showed two well-defined α-helices between residues Phe243 to Phe255 and Thr264 to Tyr277 (Figure 3). The RMSD values of the superposition of the backbone N, Cα, C atoms for N-terminal and C-terminal helices were 0.17±0.08 Å and 0.21±0.07 Å, respectively (Figure 3B, Table 1). The structured loop between the helices included residues Phe255 to Pro263. The RMSD value for the superposition of the backbone N, Cα, C atoms of the two helices was 0.38±0.10 Å, while that for all heavy atoms was 0.88±0.12 Å (Table 1). The RMSD value of superposition of backbone atoms for residues 240 to 277, for which there were sequential and long-range NOEs, was 0.59±0.20 Å, indicating that the overall structure of alpha45 was very well-defined (Figure 3C). The ribbon representations of alpha45 (Figure 3C) show that the position of the helices is slightly offset by about half a helical turn, with the C-terminal end of helix I making contact with residues of the structured loop.

### Alpha45 interacts electrostatically with SDS micelles

**Figure 4:**
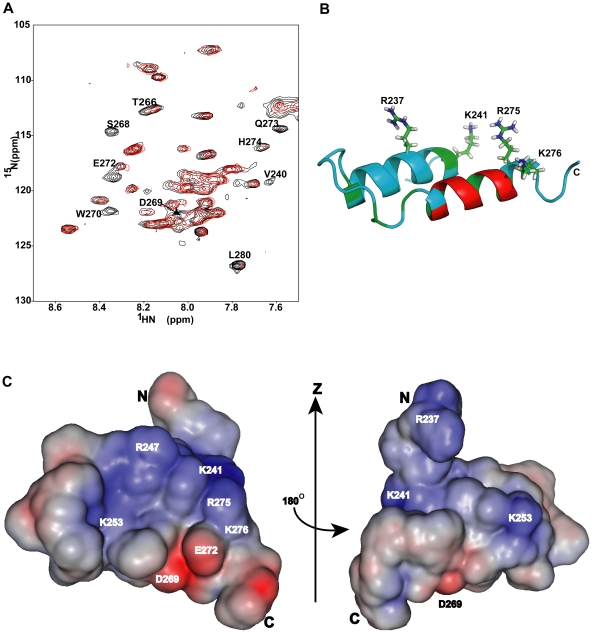
Paramagnetic effect on alpha45. **A.** Superposition of the ^1^H-^15^N HSQC spectra of alpha45 in 1% SDS (black) and in 1% SDS with 4× excess MnCl_2_ (red). Amino acids that are solvent exposed and/or accessible to Mn^2+^ had their ^1^H-^15^N signals broadened. Those amino acids are labeled. **B.** Ribbon representation of alpha45 shows the location of the basic residues that were most protected from the paramagnetic effects in 1% SDS. The regions colored in green represent the amino acids that were protected from paramagnetic effect only in SDS but not in aqueous solution. The residues whose NH signals were affected by Mn^2+^ in both SDS and aqueous solution are shown in red and found to be located on the outer surface of helix II **C.** Two views of the electrostatic map of alpha45. The blue represents basic patches and red indicates acid patches. The orientation of the left image is the same as that shown in ‘B’. The map was generated by PBEQ-solver [59], [60].

Paramagnetic studies were performed to determine how SDS might interact with alpha4 and stabilize its structure (Figure 4A). Thirty residues were observed to have their ^1^H-^15^N HSQC peaks protected from the paramagnetic effects of Mn^2+^: Arg237, Lys241, Phe243 to Tyr259, Ser261, Leu262, Thr264, Met265, Val267, Tyr271, and Val274 to Gly278. In contrast, Mn^2+^ broadened the NH signals of 10 residues that included Glu236, Val240, Thr266, Ser268 to Trp270, Glu272 to His274 and Leu280. The remaining five residues were prolines that do not have NH signals.

Interestingly, the paramagnetic protection data for alpha45 in aqueous solution revealed 19 of the 30 residues protected in the SDS sample had their signals unaffected (data not shown). This comparison showed that Lys241, Phe243, Arg247, Ser261, Leu262, Thr264, Val267, Tyr271, Val274, Arg275 and Lys276 were the only residues protected by 1% SDS. There were no amino acids whose NH signals were protected in aqueous solution but disappeared in 1% SDS.

Based on the tertiary structure of alpha45 and the pattern of paramagnetic protection, it is unlikely that the peptide was embedded into the micelles. Instead it most likely existed in a state close to its actual 3D structure. Since the NOE cross-peak patterns indicated that the N-terminal half of alpha45 was unstructured in aqueous solution, the protection of these residues was probably due to soluble protein aggregation. The residues protected only in SDS were at the N-terminal end of helix I and at the C-terminal end of helix II, and located on one surface of alpha4 (Figure 4B), indicating that their interaction with each other but more importantly with the SDS micelles helped to stabilize the secondary and tertiary structure of the peptide. Among these residues are two lysine and two arginine residues that form a basic patch on one surface of alpha45 (Figures 4B,C), suggesting that electrostatic interaction with the negatively charged surface of the SDS micelles may be important for stabilizing the helix-loop-helix structure of alpha45. Interestingly, only residues on the outer surface of helix II were most unprotected from the paramagnetic effects (Figure 4B).

### Bbox1-alpha45 binding studies

**Figure 5:**
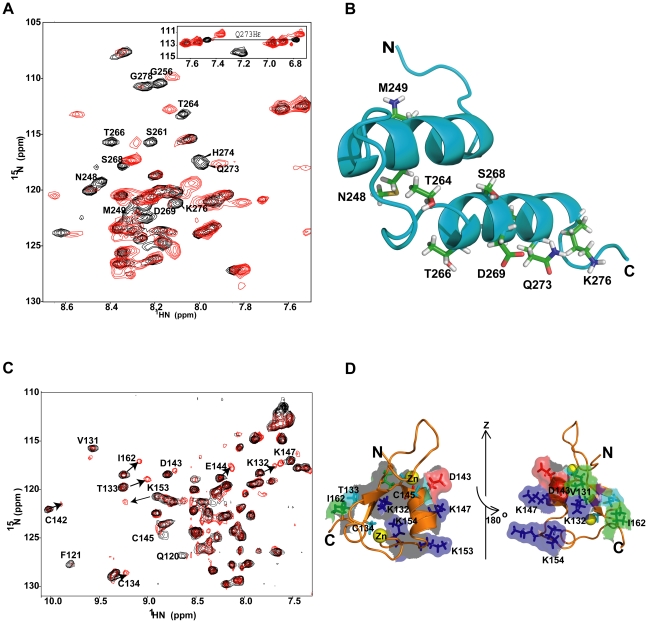
Interaction of alpha45 and the MID1 Bbox1 domain. **A.** Superposition of the ^1^H-^15^N HSQC spectra of free ^15^N-labeled alpha45 (black) and ^15^N-labeled alpha45 (red) in the presence of 1∶1 ratio of unlabeled Bbox1 in aqueous solution. Amino acids of alpha45 whose NH peaks showed chemical shift changes due to interaction are labeled. **B.** Ribbon representation of alpha45 indicates the location and residues that showed chemical shift changes when Bbox1 was added. **C.** Superposition of the ^1^H-^15^N HSQC spectra of free ^15^N-labeled Bbox1 (black) and ^15^N-labeled Bbox1 with unlabeled alpha45 (red). The ratio of Bbox1 to alpha45 was 1∶0.5. In additions to peak shifts, new peaks with intensities corresponding to ∼0.5 that of the original peak were observed at different locations, indicating Bbox1 existed in two slow exchanging states: free and bound. **D.** Surface and ribbon representations of the MID1 Bbox1 domain showing the residues that underwent chemical shift changes when alpha45 was bound. Basic and acidic residues are colored blue and red, respectively, while hydrophobic residues are colored green. Polar residues are colored cyan.

To confirm that alpha45 and the Bbox1 domain can interact, ^1^H-^15^N-HSQC spectra were acquired for ^15^N-labeled alpha45 in the presence of unlabeled Bbox1, and then for ^15^N-labeled Bbox1 in the presence of unlabeled alpha45. For alpha45, chemical shift changes indicating interaction were observed for residues Asn248, Met249, Ser261, Thr264, Thr266, Ser268, Asp269, Gln273, His274, and Lys276 (Figure 5A). With the exception of Asn248 and Met249, these residues were predicted by CSI and TALOS+ to be helical, and were indeed found to be part of helix II in 1% SDS (Figure 5B). In addition, some of these residues were also susceptible to paramagnetic effect, suggesting they were solvent exposed and on a surface distinct from the SDS interacting surface. With the exception of Asn248 and Met249, the amino acids of the N-terminal half of alpha45 did not show significant chemical shift changes, indicating that they did not interact with the Bbox1 domain. The HSQC spectrum of alpha45 in the presence of the Bbox1 domain was similar to that of alpha45 in 1% SDS, suggesting the presence of two helices.

For the Bbox1 domain, both chemical shift changes as well as the appearance of new signals were observed (Figure 5C). These observations suggested that the binding was strong, and that a subset of Bbox1 (∼40–50%) was coordinated to alpha45. The intensities of peaks corresponding to residues Lys132, Thr133, Cys142, Asp143, Glu144, Cys145, Lys147, Lys153, and Ile162 were decreased by ∼50%, consistent with ∼50% of Bbox1 forming a tight 1∶1 complex with alpha45 (Figure 5C). These residues are clustered on one surface of the Bbox1 domain (Figure 5D).

## Discussion

The NMR studies of the MID1-binding region of alpha4 reveal that the 45-amino acid portion of the C-terminus has properties of an intrinsically unstructured peptide or protein. Intrinsically unstructured proteins (IUP) do not typically adopt regular secondary or tertiary structures in solution but become structured in the presence of their binding partner (reviews in [49], [50], [51]). The IUP phenomenon is widely recognized in all organisms to play a significant role in regulating protein function and signal transduction [49], [50], [51]. For instance, the C-terminal 100 amino acids of the cell cycle regulator p27 (Kip1) are intrinsically unstructured and this affords p27 the flexibility to mediate phosphorylation and ubiquitination events of cyclin-dependent kinases [50]. Similarly, the intrinsically unstructured properties of the peptide inhibitors, I-2 and DARPP-32, of protein phosphatase1 (PP1) enable the peptides to make extensive contacts with PP1 invariant surfaces [52]. Similarly these properties could afford the C-terminus of alpha4 the flexibility and specificity to bind MID1 and PP2Ac and modulate their relative positions to each other, thereby mediating the dephosphorylation of MID1 and/or ubiquitination of PP2Ac.

The indication of the presence of α-helices by CSI and dihedral angles suggests that alpha45 may be sampling its folded-unfolded states. The addition of 1% SDS stabilizes the secondary and tertiary structure of the peptide via key interaction with two lysine and two arginine residues located at the ends of the two helices. Although this observation does not confirm that alpha4 is membrane-associated, expression of fluorescent-tagged alpha4 in COS1 cells depicted alpha4 to be localized proximal to the plasma membrane (20,28). Alpha4 appears to target PP2A that regulates the E-cadherin–β-catenin complex [21], [25]. E-cadherins are transmembrane proteins that bind β-catenin, which is part of the Wnt signaling pathway. The interaction with β-catenin is mediated by phosphorylation. It is postulated that loss of function of MID1 or alpha4 results in an increase of PP2A and an increase in the dephosphorylation of E-cadherins. As a result, cell-cell adhesion is disrupted possibly explaining structural abnormalities observed in XLOS [17], [21], [25], [26], [27].

**Figure 6:**
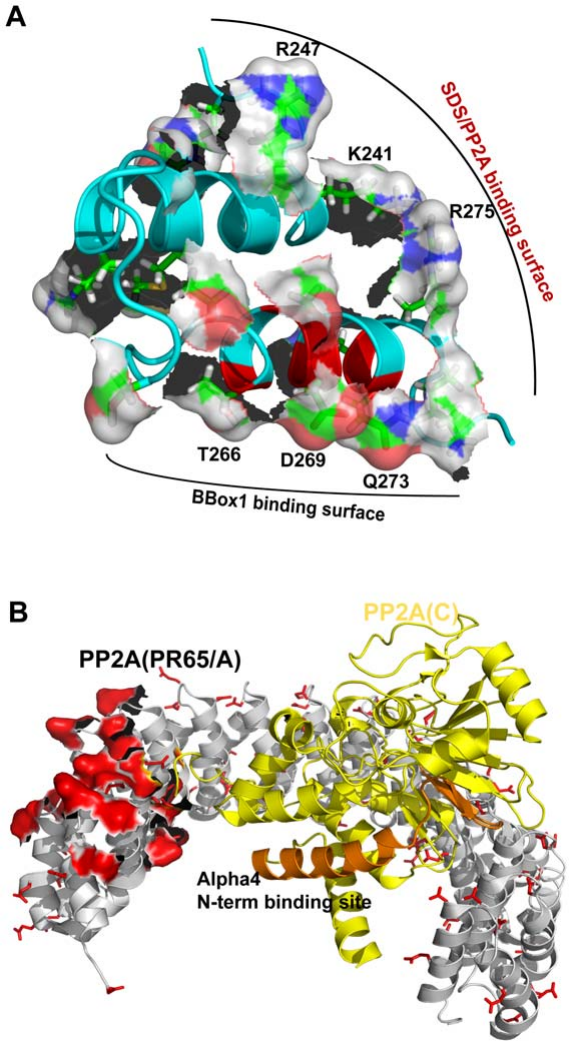
Proposed surfaces of interaction. **A.** Surface depiction of the two binding interfaces of alpha45 that could interact with the Bbox1 domain and PP2A simultaneously. **B.** Ribbon representation of the A- (scaffolding (PR65), colored gray) and C- (catalytic, colored yellow) subunits of PP2A, pdb accession code 3DW8. PR65 adopts a helix-turn-helix HEAT repeat structure. All glutamic and aspartic acids are shown in red on the A(PR65)-subunit. Located near the N-terminus of PR65 is a large negative patch, depicted by surface representation (colored red) that could accommodate the positively charged surface of alpha45. This is also the same surface on which the B- (regulatory, B55) subunit of PP2A binds. The alpha4 N-terminal binding site on PP2Ac is shown in orange.

The negatively charged head-group (SO_4_^−^) of the SDS micelles may mimic a large negatively charged surface of an alpha4 binding partner. The structure of alpha45 revealed a basic patch, consisting of Arg237, Lys241, Arg247, Arg275, and Lys276, located near the N-terminal half of helix I (Figures 4B, 6A). As it turns out, the structure of PP2A, consisting of A- (PR65), B- (regulatory) and C- (catalytic) subunits (pdb accession code 3DW8 [53]), revealed a very large negative patch on PR65 that closely mimics the negative surface of the SDS micelles (Figure 6B). In the structure depicted in Figure 6B, the B-subunit (B55), which is omitted, binds directly over the large negative surface located near the N-terminus. We postulate that the C-terminus of alpha4 can also bind this surface. Mutation of a key glutamate residue was found to disrupt alpha4 interaction [54], [55]. The helix-turn-helix structure of alpha45 also closely matches the helix-turn-helix HEAT repeats of the PR65/scaffolding subunit [56]. In contrast, the region of B55 that interacts with the large negative patch of PR65 adopts a β-sheet structure. Thus, the HEAT-like structure of alpha45 may allow the C-terminal region of alpha4 to integrate itself into the existing structure of PR65 and thus play a key role in displacing the regulatory subunit. This interaction will allow the N-terminal region of alpha4 (residues 1–221) to interact with the catalytic subunit of PP2A (PP2Ac). The key site on PP2Ac on which the N-terminal domain of alpha4 interacts involves an α-helix (colored orange on Figure 6B). This region of PP2Ac is exposed and can accommodate the N-terminus of alpha4, while the C-terminus of alpha4 can easily reach the negatively charged surface of PR65 (Figure 6B). The interaction of both the N- and C-terminal domains of alpha4 with PP2A is essential for the regulation of PP2A [31].

The residues of alpha45 that form the binding surface with Bbox1 are found along helix II and located opposite to the basic patch that would be involved in the PP2A interaction (Figures 5B, 6A). These residues can only be involved in binding the Bbox1 domain if they are part of the helix-loop-helix structure observed in 1% SDS. Thus, alpha4 undergoes a significant conformational change when Bbox1 is present. Alpha45 interacts with Lys132, Thr133, Asp143, Glu144, Lys147, Lys153 and Ile162 of Bbox1 (Figure 5D). These residues are located on one surface of Bbox1 and can easily interact with the charged and polar residues located along helix II of alpha45. Interestingly, two key lysine residues located at the C-terminal end of the helix (Lys147) and on the loop (Lys153, whose positions could not be defined in the free protein [40]), show significant chemical shift changes in the HSQC spectrum when alpha45 was added. The movement of this loop with the lysine may be key for forming the interface with alpha45. Lysine153 is next to Lys154, which is poly-ubiquitinated in MID1 [16]. In our ubiquitination studies of MID1, the addition of alpha45 significantly attenuated the autoubiquitination of the Bbox1 domain, suggesting that by binding and sequestering Lys153, alpha45 prevented or weakened the interaction of Bbox1 with the E2 conjugating enzyme [16].

In yeast two-hybrid studies probing the interaction of MID1 and alpha4, it was demonstrated that alpha4 bound Bbox1 most tightly as a single domain [57]. When the MID1 construct included Bbox2, the interaction with alpha4 decreased substantially, suggesting that Bbox2 attenuates alpha4 binding. Residues Lys132, Thr133, Ile162 of Bbox1 that form part of the binding surface with alpha45 are also involved in binding Bbox2 [58]. This observation may explain why the presence of Bbox2 could weaken the interaction between Bbox1 and alpha4 [57], [58].

In summary, the C-terminal peptide of alpha4 appears to be intrinsically unstructured but possesses the structural information that is induced upon its interaction with the Bbox1 and SDS micelles. Upon adopting a helix-turn-helix structure, the peptide maintains two distinct surfaces that could interact with both Bbox1 domain and a negatively charged surface of PP2Ac. By binding the Bbox1 domain, the RING domain of MID1, which functions as an E3 ligase, could be placed in close proximity in order to facilitate the ubiquitination of PP2Ac.
